# Automatic ML-based vestibular gait classification: examining the effects of IMU placement and gait task selection

**DOI:** 10.1186/s12984-022-01099-z

**Published:** 2022-12-01

**Authors:** Safa Jabri, Wendy Carender, Jenna Wiens, Kathleen H. Sienko

**Affiliations:** 1 Department of Mechanical Engineering, University of Michigan, Ann Arbor, MI 48109 USA; 2 Department of Otolaryngology, Michigan Medicine, Ann Arbor, MI 48109 USA; 3 Department of Electrical Engineering and Computer Science, University of Michigan, Ann Arbor, MI 48109 USA

**Keywords:** Balance, Gait, Vestibular disorders, Wearable sensors, Machine learning, Classification

## Abstract

**Background:**

Vestibular deficits can impair an individual’s ability to maintain postural and/or gaze stability. Characterizing gait abnormalities among individuals affected by vestibular deficits could help identify patients at high risk of falling and inform rehabilitation programs. Commonly used gait assessment tools rely on simple measures such as timing and visual observations of path deviations by clinicians. These simple measures may not capture subtle changes in gait kinematics. Therefore, we investigated the use of wearable inertial measurement units (IMUs) and machine learning (ML) approaches to automatically discriminate between gait patterns of individuals with vestibular deficits and age-matched controls. The goal of this study was to examine the effects of IMU placement and gait task selection on the performance of automatic vestibular gait classifiers.

**Methods:**

Thirty study participants (15 with vestibular deficits and 15 age-matched controls) participated in a single-session gait study during which they performed seven gait tasks while donning a full-body set of IMUs. Classification performance was reported in terms of area under the receiver operating characteristic curve (AUROC) scores for Random Forest models trained on data from each IMU placement for each gait task.

**Results:**

Several models were able to classify vestibular gait better than random (AUROC > 0.5), but their performance varied according to IMU placement and gait task selection. Results indicated that a single IMU placed on the left arm when walking with eyes closed resulted in the highest AUROC score for a single IMU (AUROC = 0.88 [0.84, 0.89]). Feature permutation results indicated that participants with vestibular deficits reduced their arm swing compared to age-matched controls while they walked with eyes closed.

**Conclusions:**

These findings highlighted differences in upper extremity kinematics during walking with eyes closed that were characteristic of vestibular deficits and showed evidence of the discriminative ability of IMU-based automated screening for vestibular deficits. Further research should explore the mechanisms driving arm swing differences in the vestibular population.

## Background

Vestibular disorders such as bilateral/unilateral vestibular hypofunction can impair an individual’s ability to maintain postural and/or gaze stability during standing and walking [1, 2]. The loss of vestibular function may result in symptoms of dizziness, unsteadiness and an increased risk for near-falls and falls during mobility and gait [3, 4]. Prior studies have estimated that up to 35$\%$ of Americans experience vestibular-related issues during their lifetime [1, 5, 6]. Typically, a vestibular diagnosis is determined through a battery of specialized tests (e.g., Computerized Dynamic Posturography, Videonystagmography (VNG) and Rotational Chair Testing). Access to such diagnostic tools relies, however, on the referral to specialists by primary care providers, but referral rates remain low [7], leaving affected individuals under-diagnosed.

Prior to in-depth diagnostic testing, screening tests are typically used to determine whether an individual would benefit from such specialized testing. Bedside screening tests for vestibular deficits consist of tests to assess: vestibular-ocular reflex (VOR) such as head impulse tests [8], spatial orientation such as the Fukuda Stepping test [9], and balance performance during static (standing balance) and dynamic tasks (walking balance) [10]. Additionally, the Dix-Hallpike and supine roll tests can be used to screen for Benign Paroxysmal Positional Vertigo (BPPV) [11]. However general providers may have limited knowledge and experience in performing and interpreting such tests accurately.

Clinicians typically assess balance and fall risk during gait using conventional gait assessment tools that include the Functional Gait Assessment (FGA) [12], the Dynamic Gait Index (DGI) [13], 10-Meter Walk Test [14], and Timed Up and Go (TUG) [15]. However, these conventional clinical assessments rely on timed tests and observations of path deviations that do not consider the subtle changes in full-body kinematics resulting from vestibular deficits. Observational assessments allow clinicians to examine the overall movements of individuals to assess their reactions but may not detect changes to the patterns of individual body segments during gait. Vestibular deficits have been shown to affect spatiotemporal gait parameters (such as cadence, step length [16], step width, path adherence [17], etc.) and cause gaze stability deficits, often resulting in abnormal head-trunk stabilization during stepping and walking [18]. Standard clinical gait assessment tools do not capture features related to spatiotemporal gait parameters and upper-body coordination and therefore offer limited insights into the complex kinematics of vestibular gait.

Wearable sensors such as inertial measurement units (IMUs) present an opportunity to measure and characterize gait by quantifying the movement of various body segments throughout the gait cycle. While other movement tracking technologies such as motion capture have been used in research settings, IMUs are better adapted to clinical use as they are more cost-effective, require minimal set-up, and are easily integrated into portable/wearable electronics. IMUs have been used to detect gait events and estimate spatiotemporal gait parameters such as stride length, stride time, stance time, swing time and gait speed [19], as well as to estimate upper-body kinematics [20]. Wearable IMUs have been used to estimate spatiotemporal gait metrics of individuals with vestibular deficits during the 2-Minute Walk Test [21], and measure walking/turning times during conventional gait tests such as the FGA [22] and TUG [23], thus providing a quantitative measurement of kinematic changes that occur during gait tasks when the vestibular system is affected.

Machine learning (ML) methods have further enabled the use of wearable IMUs in a variety of balance assessment and gait analysis contexts to automatically detect balance deficiencies and classify pathological gait due to Parkinson’s disease [24–28], cerebellar ataxia [29–31], and cerebral thrombosis [32]. However, few studies have applied ML methods to IMU-based kinematic data to classify and screen for gait abnormalities related to vestibular deficits. Namely, a study by Ikizoglu et al. [33] reported a binary (vestibular/control) classification model based on a dataset of kinematic data captured using IMUs placed on the feet, knees and lower back while participants walked along a 11.5 m path. Because the IMUs in this study were only placed on the lower body, abnormalities in the upper-body coordination strategies observed in populations with vestibular deficits were not captured. In addition, participants performed a single simple gait task. Another study by Nguyen et al. [34] demonstrated a binary (vestibular/control) classification model based on kinematic data from one IMU placed on the upper back while participants performed the DGI. A single IMU on the upper back was used in this study resulting in the capture of movements from one segment of the participants’ bodies during gait tasks involving head movements, stepping over an obstacle, changing speed and a pivot turn. While these studies provide evidence of the feasibility IMU-based automatic classification of vestibular gait, they investigated only a limited set of IMUs and gait tasks.

The number and placement of IMUs as well as the selection of gait tasks used to classify vestibular gait have impacts on model performance and practical implications when such systems are deployed for clinical use. Different IMU placements measure features related to the movement of different body segments and therefore capture various compensatory strategies or adaptations reported within the vestibular population such as changes to spatiotemporal gait parameters [16, 17, 35] and abnormal head-trunk stabilization [18]. The choice of IMU placement on different body segments was shown to have an effect on model performance in the context of gait-based classification of stroke and other neurological disorders [36], and the choice of IMU placement on a given body segment was shown to affect the accuracy of estimated spatio-temporal gait parameters [37, 38] and measures of stability [39]. In addition, the number of IMUs and their placement are important factors for technology adoption within clinical settings - fewer sensors and ease of placement are preferable [40]. Similarly, gait task selection can affect classification performance as different gait tasks within common functional assessments challenge participants’ sensory compensation strategies in different ways [41, 42], and therefore highlight their gait deficits under different sensory conditions. The number of tasks needed to screen for vestibular deficits also has practical implications on the duration of testing. Therefore, the goals of this study were to examine the effects of IMU placement and gait task selection on the performance of automatic vestibular gait classifiers in order to inform the design of accurate, reliable and adoptable IMU-based automatic screening tools.

## Methods

In this study, we aimed to identify wearable IMU placements and gait tasks best suited for the automatic classification of vestibular gait through ML. We used a set of full-body (including the head, trunk, arms, wrists, thighs, shanks, and feet) wearable IMUs to capture the kinematics of various body segments among participants with vestibular deficits and age-matched controls during a variety of gait tasks. We then used the kinematic data collected to extract descriptive features and train ML models to classify vestibular gait. We assessed the predictive power of models trained on features extracted from various combinations of single IMU placements and different gait tasks in terms of area under the receiver operating characteristic curve (AUROC) scores.

### Participant recruitment

Thirty study participants were recruited to participate in a single-session gait study. Fifteen of the participants were diagnosed with vestibular deficits (11 females, 4 males, 58 ± 16 y) and fifteen were age-matched controls (11 females, 4 males, 56 ± 13 y) (see Table 1). Participants were included if they had a diagnosed vestibulopathy as determined through a medical chart review by a physical therapist at the Michigan Balance Clinic, and if they were able to ambulate more than 10 m and to stand for at least 30 s without support. Participants were excluded if they scored < 24/30 on the Mini Mental State Exam (MMSE) [43], had a musculoskeletal disorder that limited their ability to walk, or had a severe visual or hearing impairment. All participants gave written informed consent. The study protocol was reviewed and approved by the University of Michigan Institutional Review Board (HUM00152737).

Data collection was conducted between May 2019 and June 2021. Nine study participants (six diagnosed with a vestibular deficit and three controls) participated in the study after March 2020 and therefore performed the gait tasks in the study protocol while wearing face masks due to COVID-19 constraints. These participants were identified in Table 1 with an asterisk (*).

### Data collection

**Fig. 1:**
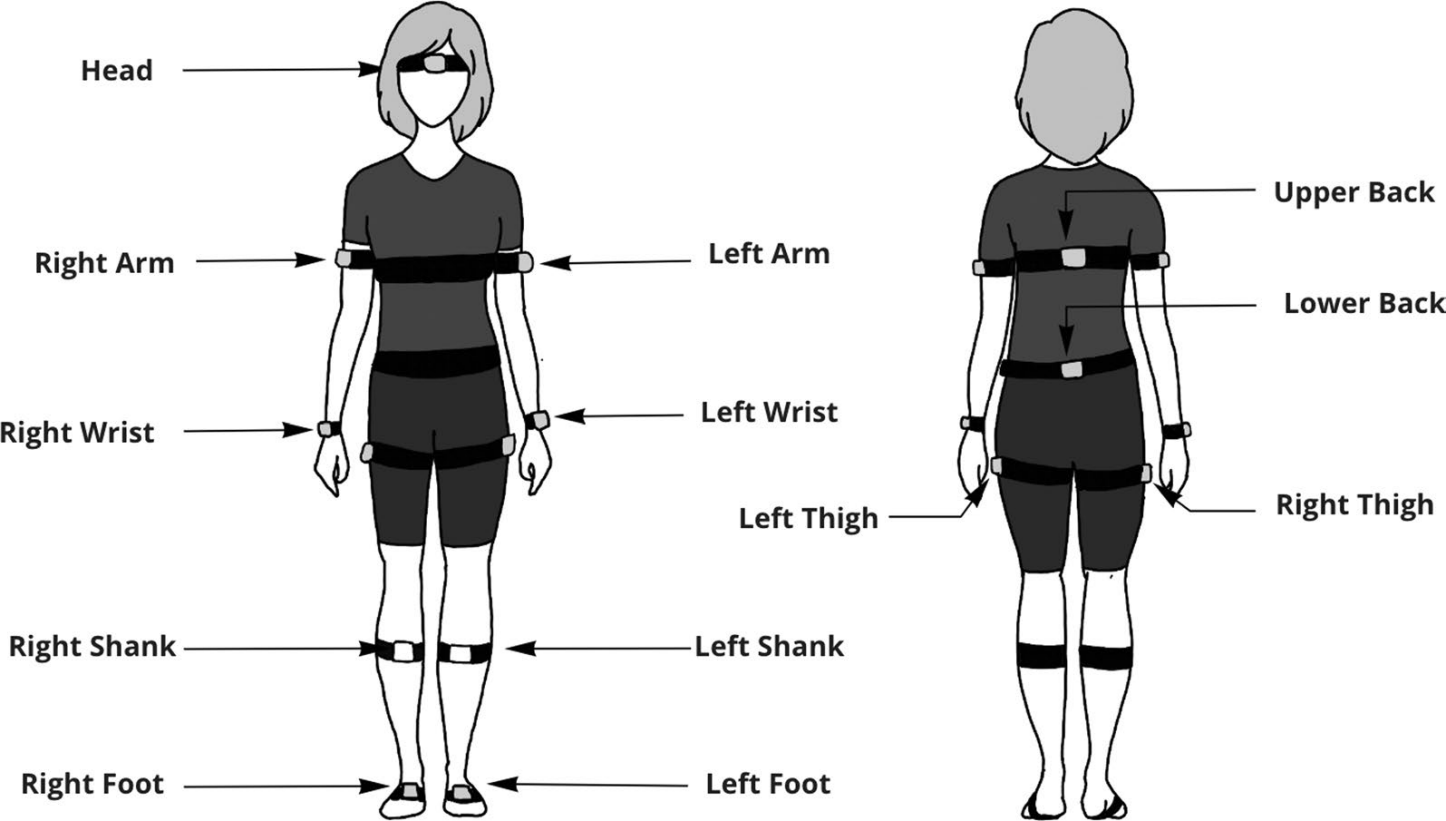
Illustration of IMU placements. Study participants wore 13 IMUs while performing gait tasks. IMUs were placed on the head, upper back, lower back, both arms, wrists, thighs, shanks, and feet

Participants wore a set of 13 wearable IMUs (Opal, APDM Inc., Portland, OR, USA) placed on the head, upper back, lower back, arms, wrists, thighs, shanks, and feet (Fig. 1). The IMUs collected synchronized tri-axial acceleration, angular rate, and magnetometer time-series measurements at a sampling rate of 128 Hz.

Participants performed a series of gait tasks along a 6 m walkway as outlined in Table 2. Tasks varying gait speed were selected based on findings from previous studies that indicated that participants with vestibular deficits showed higher variability in spatiotemporal gait parameters when walking slowly [44]. In addition, gait tasks involving vertical and horizontal head turns were included because they have been often used in vestibular rehabilitation therapy to enhance the recalibration of the vestibulo-ocular reflex (VOR) [42]. Lastly, a task involving walking with eyes closed was selected because participants with vestibular deficits have been reported to rely on somatosensory input of the lower extremities (during the acute stage) and visual cues (during the chronic stage) to compensate for the lack of vestibular input [45]. Three non-consecutive trials were performed for every gait task.

**Table 1:** Demographic information of study participants

Participant ID	Age	Sex	Diagnosis	Stage	ABC	DHI
Participant 1V	61	Female	Right Unilateral Vestibular Hypofunction (UVH)	Chronic	93	10
Participant 2V	49	Female	Right Unilateral Vestibular Hypofunction (UVH)	Sub-acute	91	14
Participant 3V	66	Female	Benign Paroxysmal Positional Vertigo (BPPV) - Unresolved	Sub-acute	81	26
Participant 4V	81	Female	Benign Paroxysmal Positional Vertigo (BPPV) - Unresolved	Chronic	96	22
Participant 5V	55	Female	Bilateral Vestibular Hypofunction (BVH)	Chronic	32	72
Participant 6V	42	Male	Right Unilateral Vestibular Hypofunction (UVH)	Chronic	88	12
Participant 7V	73	Male	Bilateral Vestibular Hypofunction (BVH)	Chronic	95	0
Participant 8V	32	Female	Bilateral Vestibular Hypofunction (BVH)	Chronic	66	34
Participant 9V	80	Male	Right Unilateral Vestibular Hypofunction (UVH)	Chronic	95	0
Participant 10V*	53	Male	Bilateral Vestibular Hypofunction (BVH)	Chronic	83	18
Participant 11V*	60	Female	Left Unilateral Vestibular Hypofunction (UVH)	Sub-acute	96	4
Participant 12V*	52	Female	Bilateral Vestibular Hypofunction (BVH)	Chronic	98	14
Participant 13V*	71	Female	Left Unilateral Vestibular Hypofunction (UVH)	Sub-acute	76	58
Participant 14V*	30	Female	Bilateral Vestibular Hypofunction (BVH)	Chronic	87	24
Participant 15V*	65	Female	Right Unilateral Vestibular Hypofunction (UVH)	Chronic	71	40
Participant 1H	50	Male	Age-matched control		96	2
Participant 2H	51	Female	Age-matched control		99	0
Participant 3H	46	Female	Age-matched control		98	0
Participant 4H	59	Female	Age-matched control		98	0
Participant 5H	68	Female	Age-matched control		97	0
Participant 6H	41	Female	Age-matched control		99	0
Participant 7H	61	Female	Age-matched control		96	0
Participant 8H	57	Female	Age-matched control		98	0
Participant 9H	64	Female	Age-matched control		96	0
Participant 10H	67	Female	Age-matched control		98	0
Participant 11H	72	Male	Age-matched control		94	0
Participant 12H	68	Male	Age-matched control		99	0
Participant 13H*	67	Female	Age-matched control		98	0
Participant 14H*	29	Male	Age-matched control		99	0
Participant 15H*	38	Female	Age-matched control		100	0

Participants were considered to have acute, sub-acute or chronic vestibular deficits if data collection was conducted in the first two weeks, between two weeks and three months, or three months after the onset of symptoms, respectively [46]

*ABC* The Activities-Specific Balance Confidence Scale, *DHI* The Dizziness Handicap Inventory

*Participants wore a face mask during the experiment following safety measures during the COVID-19 pandemic

**Table 2:** List of gait tasks performed in a 6 m walkway. Instructions adapted from the FGA [12]

Gait task	Instructions
Gait at Self-selected Speed (SSGS)	Walk at your normal speed until I tell you to stop.
Gait with Change of Speed (CGS)	Begin walking at your normal pace. When I tell you “fast” walk as fast as you can. When I tell you “slow” walk as slowly as you can.
Gait with Vertical Turns (GVHT)	Walk straight at your normal pace. After three steps tip your head up and keep walking straight. After three more steps tip your head down and keep walking. Continue alternating head movement every three steps.
Gait with Horizontal Turns (GHHT)	Walk straight at your normal pace. After three steps turn your head left and keep walking straight. After three more steps turn your head right and keep walking. Continue alternating head movement every three steps.
Gait with Eyes Closed (GEC)	Walk at your normal speed with eyes closed until I tell you to stop.
Fast Gait (FGS)	Walk as fast as you safely can until I tell you to stop.
Slow Gait (SLGS)	Walk as slowly as you can until I tell you to stop.

### Signal processing and feature extraction

#### IMU axis alignment

For each IMU, the z-axis was aligned with global gravity, the y-axis was aligned with the participant’s frontal axis and the x-axis was aligned with their sagittal axis. The z-axis was defined as the axis along which gravitational acceleration was measured during a brief period of static standing at the beginning of each trial. The y- and x- axes were determined during periods of walking by performing a principal component analysis (PCA) on the angular velocity in the two-dimensional plane orthogonal to the z-axis [47, 48]. The principal vector was assumed to be aligned with the participant’s frontal axis (y-axis) and the x-axis was its orthogonal in the two-dimensional plane.

**Table 3:** List of features extracted. For each feature category, features describing the movement of participants in each gait task were summarized by applying the corresponding statistical descriptors

Feature category	Definition	Statistical descriptor	Number of features	IMUs
Angular velocity (rad/s)	Angular velocities in x-, y-, and z-axes	Maximum, Minimum, Mean,RMS, Range	15	All 13 IMUs
Acceleration (m/s$^2$)	Acceleration in x-, y-, and z-axes	Maximum, Minimum, Mean, RMS, Range	15	All 13 IMUs
Total angular velocity (rad/s)	Square-root of the sum of squared angular velocities in x-, y- and z-axes	Maximum, Minimum, Mean, RMS, Range	5	All 13 IMUs
Total acceleration (m/s$^2$)	Square-root of the sum of squared accelerations in x-, y- and z-axes	Maximum, Minimum, Mean, RMS, Range	5	All 13 IMUs
Pitch (rad)	Angular displacement around the frontal axis perpendicular to the direction of walking (y-axis)	Mean, Range	2	All 13 IMUs
Roll (rad)	Angular displacement around the sagittal axis in the direction of walking (x-axis)	Mean, Range	2	All 13 IMUs
Area of Sway Velocity (rad$^2$/s$^2$)	Angular velocity in both pitch and roll directions	95% CI Ellipse Area (EA)	1	Head, Upper Back, Lower Back
Area of Sway (rad$^2$)	Angular displacement in both pitch and roll directions	95% CI Ellipse Area (EA)	1	Head, Upper Back, Lower Back
Stride Length (m)	Distance traveled between two footfalls of the same foot	Mean, Variance	2	Left Foot, Right Foot
Stride Time (s)	Time elapsed between two footfalls of the same foot	Mean, Variance	2	Left Foot, Right Foot
Stride Frequency (Hz)	Number of strides per second	Mean	1	Left Foot, Right Foot
Foot Speed (m/s)	Distance traveled per second	Mean	1	Left Foot, Right Foot

#### Kinematic features

For each IMU, raw acceleration and angular rate signals were pre-processed through a zero-phase bandpass filter with cutoff frequencies of 0.5-25 Hz to minimize drift and noise in the signal [36]. Angular rate signals were integrated to obtain angular positions, capturing sway throughout the gait tasks.

#### Spatiotemporal gait parameters

Raw acceleration and angular velocity signals collected from IMUs placed on the feet were processed to detect footfalls and extract spatiotemporal gait parameters. The raw velocity signals were corrected using Zero-Velocity-Updates (ZUPT) to account for integration errors. Stationary periods in the gait cycle were estimated based on acceleration, angular velocity, and stride time thresholds, then a ZUPT scheme was applied to correct for drift in the acceleration signals. The ZUPT scheme assumed that (1) the foot did not slip during footfalls and therefore $V_{footfall} = 0$ and, (2) the error or drift in the acceleration signal between two consecutive footfalls was linear. Once the acceleration signals were corrected, velocity and position signals for the foot mounted IMUs were obtained through integration allowing foot speed, stride length/time, and stride frequency to be computed. The kinematic and spatiotemporal gait features extracted from each IMU placement are described in Table 3. Features included ones that have been previously reported to show differences between gait patterns from individuals with vestibular deficits and controls [33, 34] as well as features commonly used to quantify balance performance during gait.

### Datasets

For each trial of each type of gait task performed (Table 2), a dataset of features and corresponding labels was created. Each gait trial for each study participant represented a row (30 participants $\times$ 3 trials = 90 rows) with 590 features extracted from the data collected via IMUs and one label corresponding to the diagnosis of the study participant (1 if vestibular or 0 if age-matched control). For each of the seven gait tasks performed, a separate dataset was created on which to train and test ML models.

### Machine learning

**Fig. 2:**
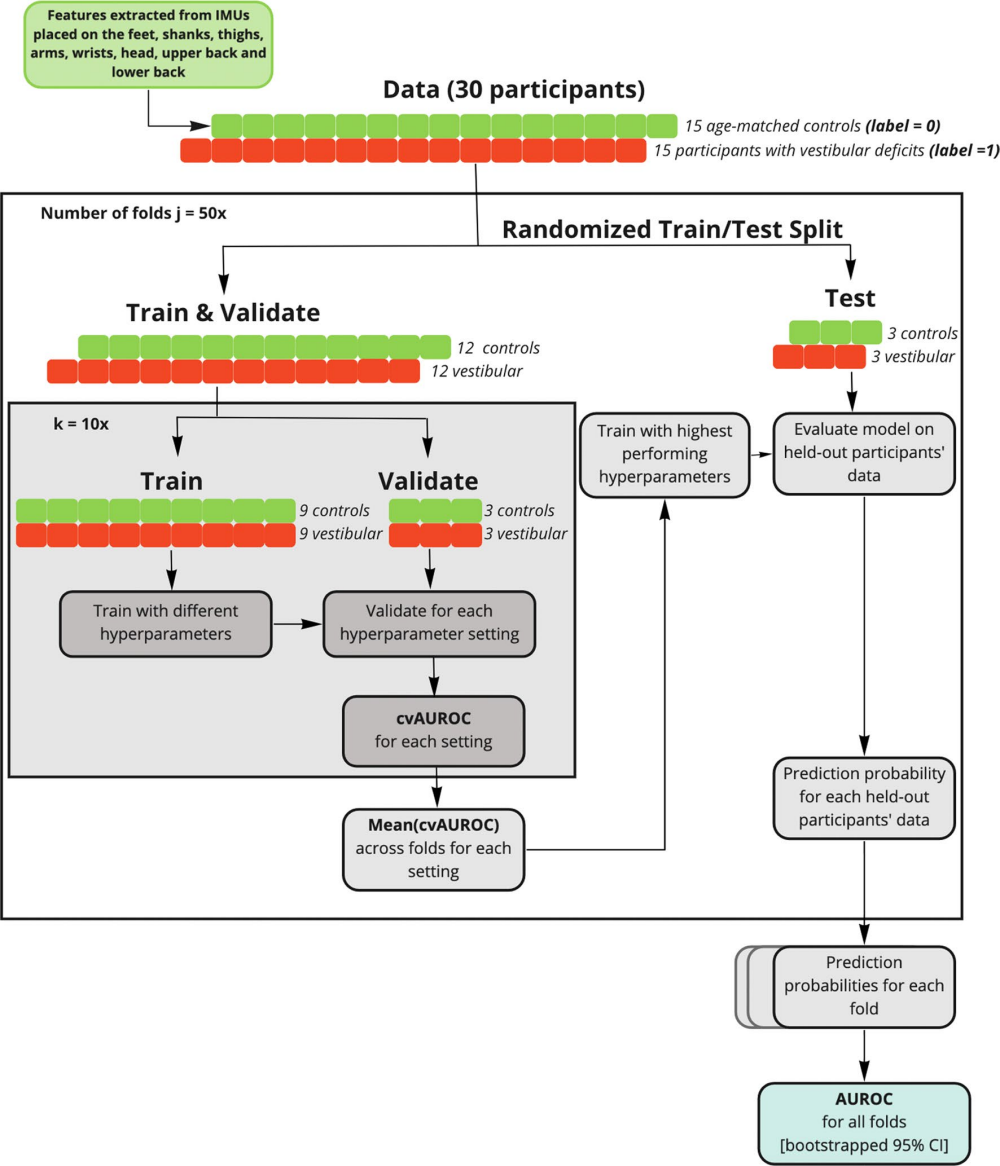
Overview of model training and evaluation scheme. A Random Forest (RF) classifier was trained to predict participants’ diagnoses based on kinematic features extracted from the IMUs. For each fold, the datasets were split into a training set (24 study participants) and a testing set (six study participants) such that both classes were evenly represented. Classification performance was assessed by calculating the AUROC score for the merged predictions on held-out test sets

Given the feature vectors for a trial/task, we trained a Random Forest classifier to classify gait trials from participants with vestibular deficits and age-matched controls. We repeatedly split the data 80/20 train/test 50 times such that the data for six study participants at a time (three with vestibular deficits and three controls) were selected to be part of the testing set (Fig. 2). This stratified split based on participants eliminates the possibility of a model learning individual characteristics of a participant’s gait instead of the underlying commonalities characteristic of vestibular gait. Hyperparameters (such as the number of estimators, the maximum number of features, the maximum depth, and the minimum number of samples per leaf) were selected based on training data using a group k-fold cross validation scheme, optimizing for the area under the receiver operating characteristic curve (AUROC) scores on the validation data.

To determine the effects of IMU placement and gait task selection for a single IMU on classification performance, models were trained on data from one IMU at a time (i.e., 44 features for IMUs attached to the upper limbs and legs, 46 features for the head and trunk, and 50 features for feet were included in each model, see Table 3) for each task, resulting in 7 IMUs $\times$ 7 tasks = 49 models. Features introduced in each model were standardized such that $\mu = 0$, $\sigma = 1$ to avoid introducing bias in the model due to the different measurement scales used.

### Model evaluation

For each model, we evaluated performance across all 50 test sets. We report the overall AUROC score across all test folds with bootstrapped 95$\%$ confidence intervals (CI) by merging the instances from all folds together by their assigned prediction probability scores into one large test set [49] (Fig. 2). Such an evaluation relies on having good model calibration.

To examine the effects of IMU placement and gait task selection on model performance, we calculated the average AUROC score of each IMU placement, averaged across gait tasks, and the average AUROC score of each task, averaged across IMU placements.

The model with the highest test AUROC score for a given IMU placement and a given gait task was identified as the highest performing single-IMU model. In addition, the accuracy, F-1 score, specificity, sensitivity and confusion matrix assuming a classification threshold of 0.50 were reported for this highest performing single-IMU model. Feature permutation importance analysis was performed to identify kinematic feature clusters driving classification performance. To account for multi-collinearity between the kinematic features introduced to the model, we calculated pairwise Pearson’s correlations for features and identified feature groups or clusters using Ward’s method for hierarchical clustering. Feature permutation performance was calculated by evaluating the decrease in each model’s test AUROC scores when the values of each feature cluster were shuffled. Feature clusters were ranked according to the observed drop in test AUROC scores.

An independent Welch t-test analysis was then performed on the features in the most important cluster to examine the directionality of the differences captured through these features among the two classes (vestibular and age-matched controls).

## Results

Test AUROC scores based on data collected from each IMU are reported in Table 4 for each of the seven gait tasks performed. Several models were able to predict vestibular diagnosis better than a random assignment (AUROC > 0.5). IMU placement had an effect on model performance. An IMU mounted on the left arm achieved the highest mean performance across all gait tasks (AUROC = 0.76 [0.68, 0.88]).

Gait task selection also had an effect on model performance. The highest mean performance across all IMUs for a given gait task (AUROC = 0.74 [0.61, 0.88]) was achieved when the classifier was built with data collected while participants walked with eyes closed.

**Fig. 3:**
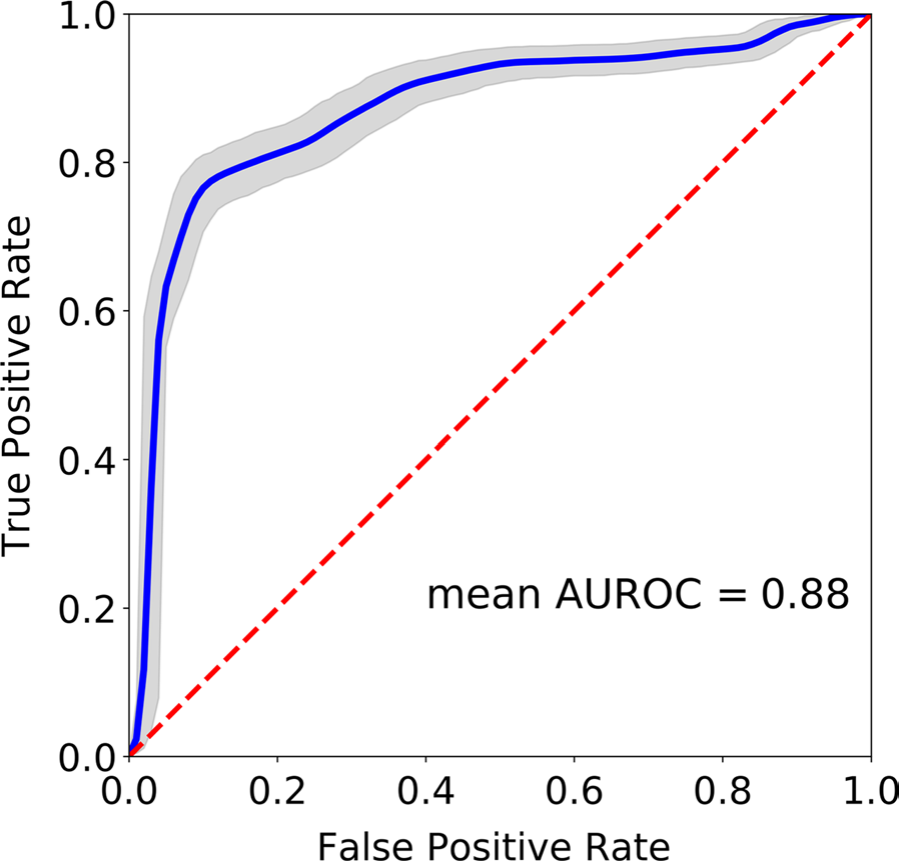
Receiver-Operating Characteristic (ROC) curve for the highest performing single-IMU model. The model based on data from the left arm during gait with eyes closed achieved an average AUROC score of 0.88 [0.84, 0.89]

The highest classification performance overall (AUROC = 0.88 [0.84, 0.89]) was achieved when predicting study participants’ diagnoses based on features extracted from the IMU placed on the left arm while they performed the walking with eyes closed task. The receiver operating characteristic (ROC) curve (Fig. 3) reflects the trade-off between sensitivity and specificity in this model. For a classification threshold of 0.50, this model had an accuracy of $80\%$ $[78\%, 83\%]$ and an F-1 score of 0.81 [0.78, 0.83]. The confusion matrix for this model is reported in Table 5.

**Table 4:**
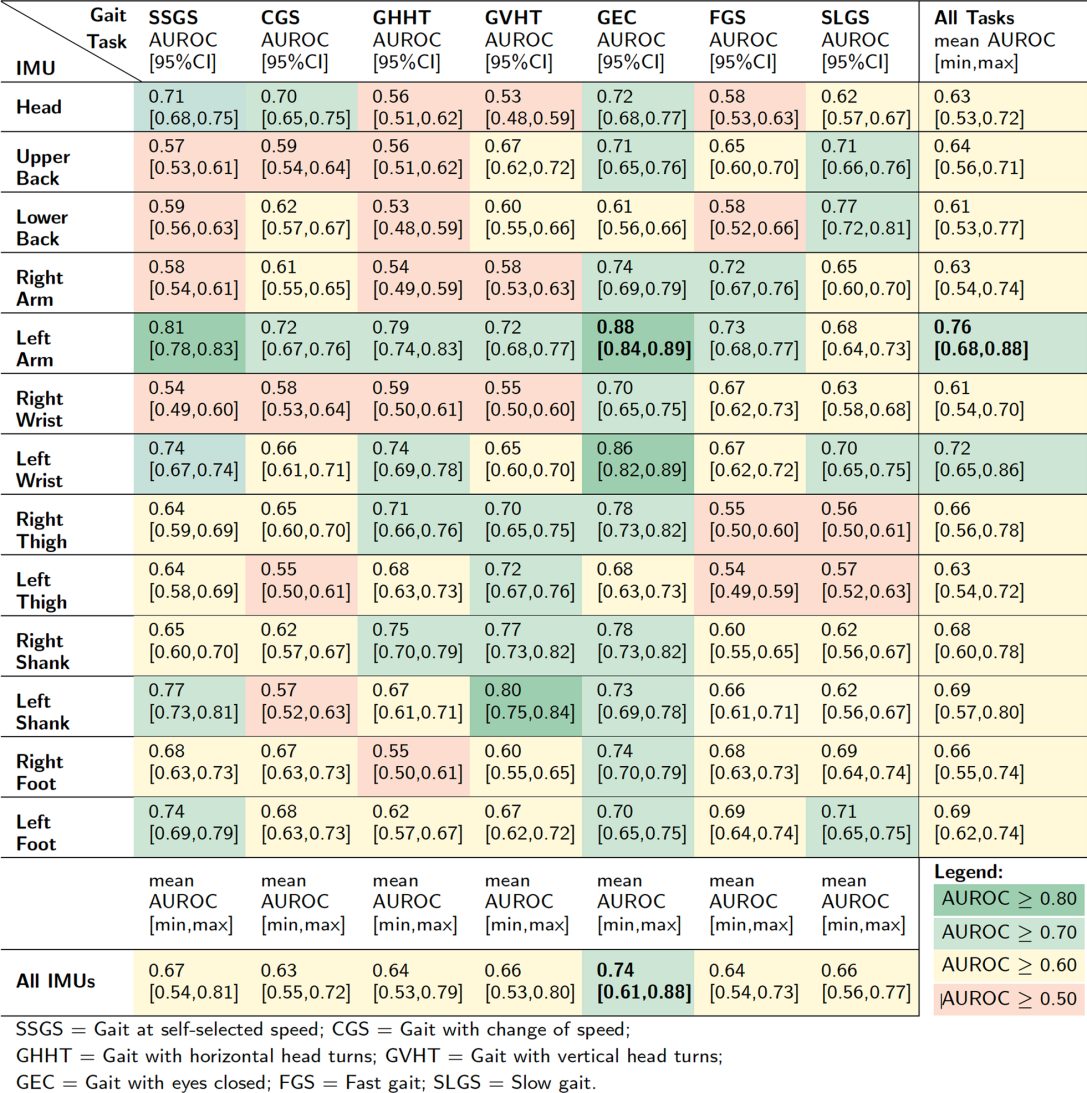
Classification results: Test AUROC scores and 95% CI for each IMU placement and gait task performed

**Table 5:**
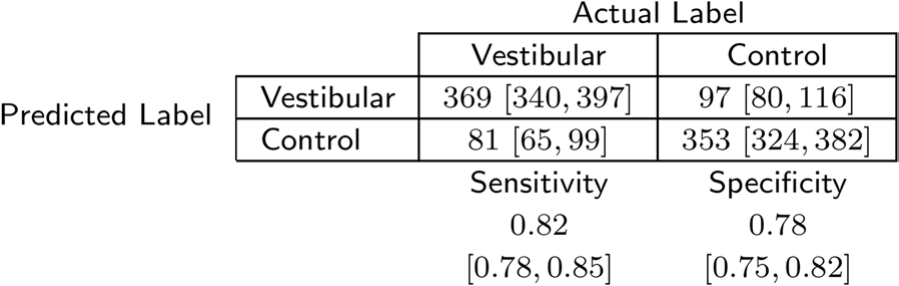
Confusion matrix [95$\%$ CI] for the highest performing single-IMU model trained on kinematic data collected from the left arm during walking with eyes closed. Predicted labels were defined with a 0.5 prediction threshold

**Fig. 4:**
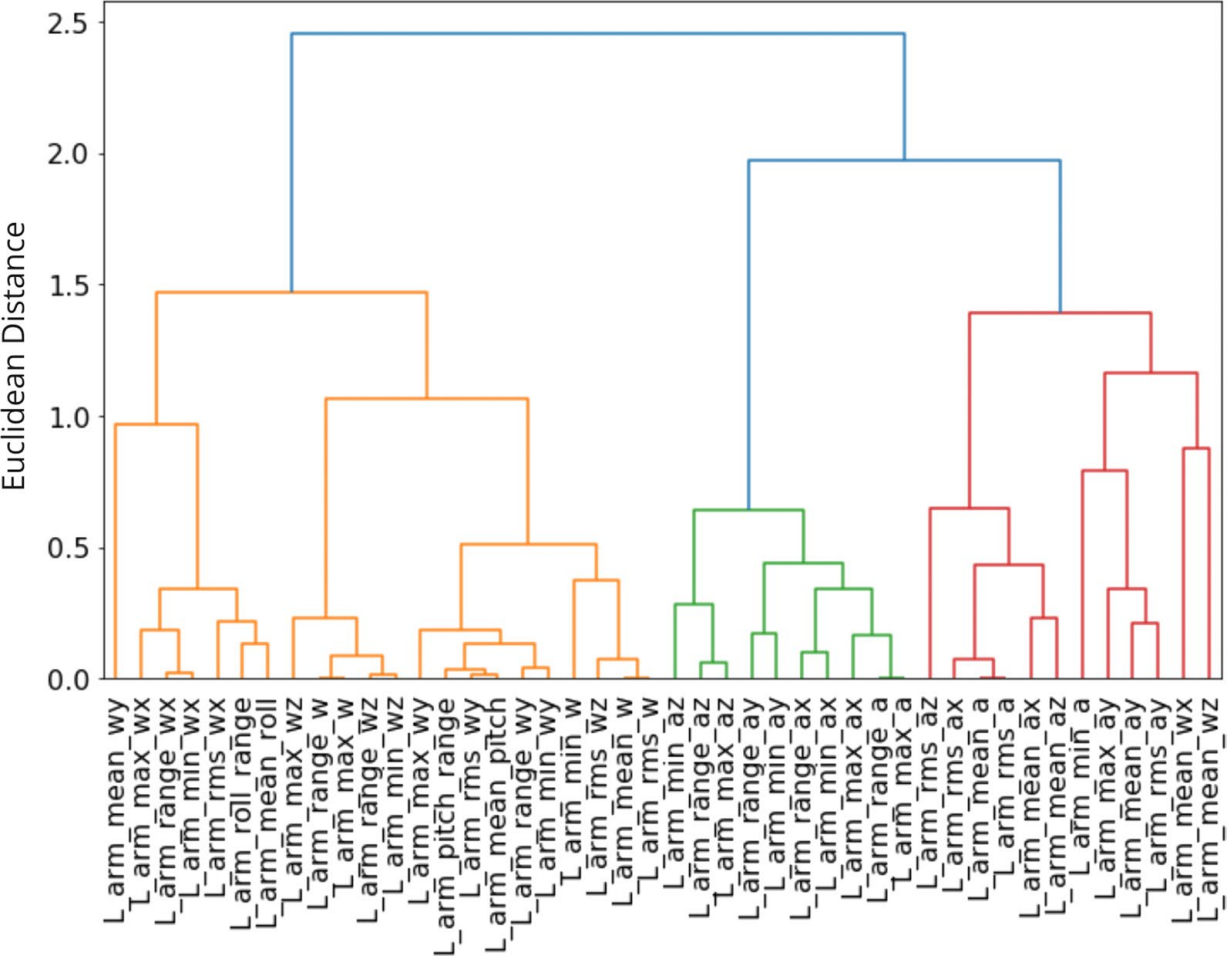
Correlation-based feature clusters. Dendrogram showing hierarchical clustering of correlated features according to Ward’s method. Three main feature clusters were identified (shown left to right): the first cluster included angular velocities and displacements, the second included accelerations, and the third included a combination of both accelerations and angular velocities

Based on the feature correlation analysis and Ward’s hierarchical clustering (Fig. 4), we identified three main feature clusters as outlined in Table 6. Features from cluster 1, related to angular velocities and angular displacements, had the greatest impact on model performance. Clusters 2 and 3 included features related to accelerations and were not associated with significant changes in model performance.

To examine the directionality of the differences between the two classes (vestibular and age-matched controls) based on the features that emerged as important from the feature permutation importance analysis, independent Welch t-test results for features included in cluster 1 are outlined in Table 7.

**Table 6:** Feature permutation importance results for the highest performing single-IMU model trained on kinematic data collected from the left arm during walking with eyes closed

Feature cluster	Features	AUROC drop [95% CI]
Cluster 1	Range of angular velocity ($\omega _{total}$,$\omega _{x}$,$\omega _{y}$,$\omega _{z}$)	0.25 [0.20, 0.29]
	Maximum angular velocity ($\omega _{total}$,$\omega _{x}$,$\omega _{y}$,$\omega _{z}$)	
	Minimum angular velocity ($\omega _{total}$,$\omega _{x}$,$\omega _{y}$,$\omega _{z}$)	
	Mean angular velocity ($\omega _{total}$,$\omega _{y}$)	
	RMS angular velocity ($\omega _{total}$,$\omega _{x}$,$\omega _{y}$,$\omega _{z}$)	
	Range of pitch angular displacement ($\theta _{y}$)	
	Range of roll angular displacement ($\theta _{x}$)	
	Mean of pitch angular displacement ($\theta _{y}$)	
	Mean of roll angular displacement($\theta _{x}$)	
Cluster 2	Range of acceleration ($a_{total}$,$a_{y}$,$a_{y}$,$a_{z}$)	0.01 [0.00, 0.01]
	Maximum acceleration ($a_{total}$,$a_{y}$,$a_{z}$)	
	Minimum acceleration ($a_{y}$,$a_{y}$,$a_{z}$)	
Cluster 3	Maximum acceleration ($a_{y}$)	0.01 [0.00, 0.01]
	Minimum acceleration ($a_{total}$)	
	Mean acceleration ($a_{total}$,$a_{y}$,$a_{y}$,$a_{z}$)	
	RMS acceleration ($a_{total}$,$a_{y}$,$a_{y}$,$a_{z}$)	
	Mean angular velocity ($\omega _{y}$,$\omega _{z}$)	

**Fig. 5:**
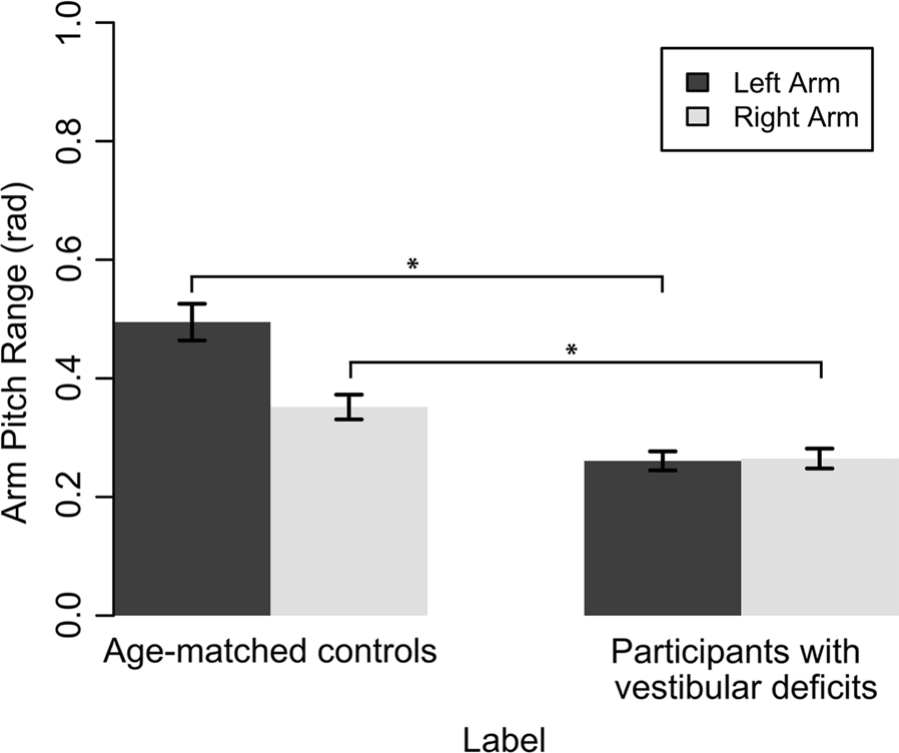
Mean range of arm pitch displacement for participants with vestibular deficits and age-matched controls. Error bars show standard error of the mean. On average, participants with vestibular deficits (M = 0.26, SD = 0.11) had decreased left arm pitch ranges of angular displacement while walking with eyes closed compared to age-matched controls (M = 0.49, SD = 0.21). The difference was statistically significant t(66) = 6.72, p < 0.05 with a large effect size r = 0.64. For the right side, participants with vestibular deficits (M = 0.26, SD = 0.11) had decreased arm pitch ranges of angular displacement while walking with eyes closed when compared to age-matched controls (M = 0.35, SD = 0.14). While the difference was statistically significant t(84)= 3.24, p < 0.05, the effect size was smaller r = 0.37

Compared to age-matched controls, participants with vestibular deficits showed a decrease in left arm angular velocity and range of angular displacement. A similar but less pronounced trend was observed for the right arm (Fig. 5). Notably, the range of angular displacement in the roll direction was not significantly different between the two classes (vestibular and age-matched controls).

**Table 7:** Independent Welch t-test results for features from cluster 1. Cluster 1 was identified as the group of features with the highest impact on classification performance of vestibular gait based on kinematic data from an IMU mounted on the left arm during walking with eyes closed

Feature	Age-matched controls	Participants with vestibular deficits	t-value	p-value	Effect size r
Range of angular velocity ($\omega _{total}$)	M = 3.19 SD = 0.78	M = 2.36 SD = 1.12	t(78.3) = 9.04	< 0.05	0.42
Maximum angular velocity ($\omega _{total}$)	M = 3.30 SD = 0.80	M = 2.40 SD = 1.12	t(80.1) = 4.29	< 0.05	0.43
Minimum angular velocity ($\omega _{total}$)	M = 0.09 SD = 0.06	M = 0.04 SD = 0.03	t(60.0) = 4.99	< 0.05	0.43
Mean angular velocity ($\omega _{total}$)	M = 1.27 SD = 0.36	M = 0.67 SD = 0.26	t(79.7) = 9.04	< 0.05	0.71
RMS angular velocity ($\omega _{total}$)	M = 1.43 SD = 0.39	M = 0.78 SD = 0.28	t(80.1) = 9.11	< 0.05	0.71
Range of pitch angular displacement ($\theta _{y}$)	M = 0.49 SD = 0.21	M = 0.26 SD = 0.11	t(66.0) = 6.71	< 0.05	0.64
Range of roll angular displacement ($\theta _{x}$)	M = 0.13 SD = 0.06	M = 0.13 SD = 0.06	t(87.8) = -0.17	0.79	-
Mean of pitch angular displacement ($\theta _{y}$)	M = 0.11 SD = 0.05	M = 0.05 SD = 0.03	t(64.0) = 7.65	< 0.05	0.69
Mean of roll angular displacement($\theta _{x}$)	M = 0.02 SD = 0.05	M = 0.02 SD = 0.03	t(87.5) = 0.85	0.39	-

## Discussion

This study trained ML models to automatically classify vestibular gait based on kinematic IMU data and examined the effects of IMU placement and gait task selection on classification performance. The models presented in this study were able to learn to accurately classify participants with vestibular deficits based on kinematic data collected with wearable IMU sensors during a variety of gait tasks.

IMU placement affected model classification performance. An IMU on the left arm enabled better classification of participants with vestibular deficits and age-matched controls across all gait tasks with a mean AUROC of 0.76 [0.68, 0.88]. Other IMU placements also achieved good classification performance for some of the gait tasks (AUROC $\ge$ 0.70) (Table 4). Previous studies have reported ML-based classification models based on kinematic data collected from IMUs placed on the lower limbs and/or trunk during gait for participants with vestibular deficits [33, 34], but they have not explored upper extremity placements of IMUs. In a previous study examining the effect of IMU placement on the classification of stroke and other neurological disorders [36], a shank IMU placement resulted in better model performance compared with feet, thighs, and lower back placements. Our results (Table 4) also showed that on average, placing an IMU on the left or right shank resulted in similar or slightly higher performance than placing IMUs on other lower-body segments.

Gait task selection also affected classification performance. The walking with eyes closed gait task was best able to discriminate between participants with vestibular deficits and age-matched controls across all IMU placements with a mean AUROC of 0.74 [0.61, 0.88]. This finding agrees with previous studies that have described increased deficits in vestibular gait when individuals lacked visual input [16, 22, 50]. In addition, prior studies have also indicated differences between individuals with vestibular deficits and controls when they walked and turned their heads [42, 50, 51] or walked slowly [35, 44, 52]. However, we found that walking with eyes closed enabled better discrimination between the two groups. The models trained on IMU data from other gait tasks were able to achieve good classification performance (AUROC $\ge$ 0.70) for some IMU placements (Table 4), but walking with eyes closed yielded a more consistent classification performance across IMU placements.

The best preforming model across all IMU placements and gait tasks achieved an AUROC score of 0.88 [0.84, 0.89] based on IMU data collected from the left arm during walking with eyes closed. Specifically, participants with vestibular deficits had reduced left arm angular velocities and pitch angular displacements when they walked with eyes closed. This classification performance was aligned with scores reported by Ikizoglu et al. ranging from 0.82 to 0.86 for a similar ML-based binary classification for gait patterns from individuals vestibular deficits and controls based on kinematic data extracted from lower-body IMUs [33]. A recent study by Grove et al. [21] examining the differences between gait patterns from individuals vestibular deficits and controls during the 2-Minute Walk Test using statistical methods reported AUROCs of 0.80 and 0.79 based on right and left stride lengths, respectively, and an AUROC of 0.86 for peak turn velocity. In a different ML-based study by Nguyen et al. [34], higher test AUROC scores (AUROC = 0.98) were achieved when classifying vestibular gait. Notably, in the Nguyen study, data from the same participants were included in both the training and testing sets. By holding out all instances of a subset of our participants’ data for model evaluation, we were able to assess classification performance on data from individuals the model had not trained on to mimic real-world classification problems. In a recent study, Grove et al. [22] proposed the Gait Disorientation Test as new method to screen for vestibular deficits, based on the difference between the time it takes individuals to complete a 6 m walk with eyes open at self-selected speed and eyes closed. The Gait Disorientation Test had an AUROC score of 0.91 [0.82, 1.0] on an initial dataset and AUROC = 0.89 [0.78, 1.0] on an external test set. Again, this performance was consistent with the performance achieved with our models. While the Gait Disorientation Test presents advantages in terms of cost and simplicity, the models described in our study provide additional insights into the characteristic movements of individuals with vestibular deficits during gait.

Based on the features extracted from the IMU placed on the left arm, participants diagnosed with vestibular deficits tended to reduce their left arm swing compared to age-matched controls as reflected through their lower range, maximum, minimum, mean, and RMS angular velocities, and reduced range and mean pitch angular displacements. A similar but less pronounced trend was also observed on the right arm whereby participants with vestibular deficits had reduced arm swing on both sides compared to age-matched controls.

Reduced arm swing has been reported in literature as a sign of cautious gait among healthy older adults when compared to younger adults [53, 54]. In general, left-dominant arm swing is common among healthy adults regardless of handedness [55]. However, the effect of the side of the vestibular deficit (i.e., left, right) for participants with unilateral vestibular hypofunction (UVH) was not examined in this study due to the limited sample size of participants with left UVH (Table 1). Prior gait studies have indicated a tendency to increase plantar pressure towards the side of the lesion in participants with unilateral vestibular neuritis and vestibular schwannoma, especially during walking with eyes closed [56, 57], suggesting that a relationship between the side of lesion and arm swing kinematics is possible.

Arm swing has been reported to be positively correlated with gait speed in healthy adults [54]. It is therefore possible that the reduction in arm swing observed in participants with vestibular deficits was associated with the decrease in their gait speed relative to age-matched controls when walking with eyes closed. In our study, gait speed was estimated through the foot-mounted IMUs and therefore was included as a feature in models based on foot-mounted IMUs. As shown in Table 4, the model using data from a left arm IMU outperformed models using data from foot-mounted IMUs for the same task, indicating that arm swing features may have captured more salient differences between participants with vestibular deficits and age-matched controls.

Changes in arm swing kinematics have been reported in prior gait studies examining the effects of cognitive load and movement disorders. A study by Killeen et al. [58] reported a unilateral reduction in arm swing on the right side during dual-task gait in healthy participants, indicating a possible relationship between cognitive load and arm swing during gait. In addition, arm swing amplitude and asymmetry have been reported as early signs of other pathologies affecting gait performance such as Parkinson’s Disease [59–61]. Further investigation into arm swing kinematics during gait among individuals with vestibular pathologies is needed to better understand the differences captured in our study.

Furthermore, participants with vestibular deficits showed differences in their upper back and head kinematics when walking with eyes closed when compared to age-matched controls, allowing models trained on data from an IMU mounted on the head and the upper back to achieve AUROC scores $\ge$ 0.70. Findings from a recent study by Zobeiri et al. [62] indicated that participants with chronic unilateral vestibular hypofunction following vestibular schwannoma surgical resection had a statistically significant decrease in their head pitch range of motion when walking with eyes closed. In addition, a review paper by Han et al. [42] described compensatory strategies employed by participants with vestibular lesions that aim to decrease head, neck and trunk rotations to reduce head movements. Previous studies described attempts to “lock-down” the head to the trunk among individuals with vestibular loss through co-contractions of the neck muscles in response to small postural perturbations to the body [63]. Similar adaptation strategies have been described in the context of the development of independent walking in children [64], age-related gait changes observed in older adults [65], and locomotion post long-duration spaceflight [66].

The models trained on data extracted from IMUs mounted to the lower limbs (thighs, shanks, and feet) while participants walked with eyes closed were able to achieve AUROC scores $\ge$ 0.70 (with the exception of the IMU mounted to the left thigh). This finding indicated differences between gait patterns from individuals with vestibular deficits and age-matched controls based on lower-body kinematics and agreed with results from previous studies [22, 34], which indicated that individuals with vestibular deficits walked more slowly when performing a walking with eyes closed task.

The binary classification approach used in this study was able to classify vestibulopathic and age-matched control gait, but it did not account for other gait disorders. A multi-class approach could be used to examine the possibility of differentiating between vestibular and other sensory-related gait deficits. A second limitation relates to the introduction of face masks for participants who contributed to the study during the ongoing COVID-19 pandemic. New studies have examined the effect of face masks on individuals’ abilities to detect and avoid obstacles and highlighted the added restrictions to their lower visual fields [67]. The introduction of face masks in our study may have introduced an additional challenge to participants who may have been more visually reliant. This limitation, however, would not have affected participants during the walking with eyes closed gait task since all participants were deprived of visual input.

Overall, this study examined the effect of IMU placement and gait task selection on the automatic detection of gait deficits due to vestibular deficits. By using only one IMU on the left arm while study participants walked with their eyes closed, the models developed in this study were able to identify 82$\%$ of participants with a vestibular diagnosis while screening out 78$\%$ of age-matched controls. This finding may have practical implications on the feasibility and usability of IMU-based automatic screening tools for vestibular deficits, as an arm or wrist placement is more convenient and less-obtrusive than a head, trunk, or lower body placement.

## Data Availability

The datasets used and/or analyzed during the current study are available from the corresponding author on reasonable request.

## Abbreviations

ML: Machine learning
IMU: Inertial measurement unit
DGI: Dynamic Gait Index
FGA: Functional Gait Assessment
TUG: Timed Up and Go
ABC: Activities-specific Balance Confidence
DHI: Dizziness Handicap Inventory
SSGS: Self-selected gait speed
CGS: Change of gait speed
GVHT: Gait with vertical head turns
GHHT: Gait with horizontal head turns
GEC: Gait with eyes closed
FGS: Fast gait speed
SLGS: Slow gait speed
RMS: Root-mean-square
ROC: Receiver operating characteristic curve
AUROC: Area under the operating characteristic curve
CI: Confidence interval
